# Research on time series prediction of multi-process based on deep learning

**DOI:** 10.1038/s41598-024-53762-1

**Published:** 2024-02-14

**Authors:** Huali Zheng, Yu Cao, Dong Sun, Mingjun Wang, Binglong Yan, Chunming Ye

**Affiliations:** 1 China Tobacco Zhejiang Industry Co., LTD, Hangzhou, China; 2 Business School, University of Shanghai for Science and Technology, Shanghai, China

**Keywords:** Deep reinforcement learning, Combinational prediction, D3QN algorithm, Neural network, Time series prediction, Computational science, Computer science, Information technology, Software

## Abstract

Aiming at the problem of data fluctuation in multi-process production, a Soft Update Dueling Double Deep Q-learning (SU-D3QN) network combined with soft update strategy is proposed. Based on this, a time series combination forecasting model SU-D3QN-G is proposed. Firstly, based on production data, Gate Recurrent Unit (GRU) is used for prediction. Secondly, based on the model, SU-D3QN algorithm is used to learn and add bias to it, and the prediction results of GRU are corrected, so that the prediction value of each time node fits in the direction of reducing the absolute error. Thirdly, experiments were carried out on the dataset of a company. The data sets of four indicators, namely, the outlet temperature of drying silk, the loose moisture return water, the outlet temperature of feeding leaves and the inlet water of leaf silk warming and humidification, are selected, and more than 1000 real production data are divided into training set, inspection set and test set according to the ratio of 6:2:2. The experimental results show that the SU-D3QN-G combined time series prediction model has a great improvement compared with GRU, LSTM and ARIMA, and the MSE index is reduced by 0.846–23.930%, 5.132–36.920% and 10.606–70.714%, respectively. The RMSE index is reduced by 0.605–10.118%, 2.484–14.542% and 5.314–30.659%. The MAE index is reduced by 3.078–15.678%, 7.94–15.974% and 6.860–49.820%. The MAPE index is reduced by 3.098–15.700%, 7.98–16.395% and 7.143–50.000%.

## Introduction

In a continuous production process, the silk production link plays a vital role in production quality^1^. The processes of loose moisture return and silk drying in the silk making link mainly improve the processability of raw materials by adjusting the temperature and moisture content, so that the final products have better taste and better quality^2^. Therefore, accurate prediction of the temperature and moisture content of each process in the silk production link, so as to improve the control accuracy in the multi-process, has always been an important research direction of enterprises.

The collection data of the silk production link are typical time series data^3^. At present, a lot of research has been carried out on time series data prediction methods at home and abroad. Geng et al.^4^ proposed a random forest optimization model fused with Monte Carlo to improve the learning accuracy of small sample data, aiming at the problem of low prediction accuracy of small sample data. Zhao et al.^5^ proposed the SSA-LSTM model to solve the problem of non-stationarity and complexity of power compliance data, and used the sparrow search algorithm to optimize the parameters of LSTM. The results show that the model can effectively improve the prediction accuracy. Yao et al.^6^ proposed a GRU algorithm with attention mechanism to predict the remaining life of rolling bearings. The results show that the model has better performance in pro-cessing sequence data and is more suitable for the remaining life prediction of rolling bearings. Liu et al.^7^ proposed the DQN-L-G-B combined prediction model based on deep Q-learning, which effectively completed the accurate prediction of soil moisture and temperature in the cultivated layer based on soil near-surface air temperature and humidity.

Most research on the prediction of leaves production focuses on the optimization and expansion of PID control method^8^. The model of this method is simple and easy to implement^9^, but affected by system time delay, nonlinearity and other factors, it is prone to problems such as regulation lag and low control accuracy^10^. In order to improve the control level, relevant researchers are also actively exploring the feasibility of big data, artificial intelligence and other technologies in this field. Tang Jun et al.^11^ adopted Bayesian network analysis method to establish a complex model between process parameters and quality indicators of loose moisture return, which improved the quality prediction accuracy of this process. Yu et al.^12^ used deep reinforcement learning to predict the temperature of silk drying in the non-steady state process of the material head in the silk making and silk drying process, and verified the feasibility of the results through simulation tests. Yin et al.^13^ proposed a prediction method combining Seq2Seq and time series attention mechanism, which provided a method and implementation approach for the accurate prediction of process manufacturing process quality with multi-process coupling. As there are many interference factors in the production process of leaf wire making, and the data collected by the machine have strong nonlinearity, uncertainty and lag^11^, the existing methods still have large room for improvement in prediction ac-curacy and stability.

In view of this, this paper proposes a time series combined prediction model SU-D3QN-G based on Soft Update Dueling Double Deep Q-learning algorithm with soft update strategy. Firstly, the GRU model, which is good at capturing the dynamic change law in time series data, was used to predict four groups of data in the silk production process, including the outlet temperature of drying silk, the loose moisture return water, the outlet temperature of feeding leaves and the outlet water of leaf silk heating and humidifying. Then, the SU-D3QN algorithm is used to learn and fit the bias of each time node, which further improves the accuracy and stability of the prediction of production process parameters, so as to provide more accurate guidance for improving the control stability of the temperature and moisture content of the silk making link.

## Overview of related algorithms

### Gated recurrent unit

**Figure 1:**
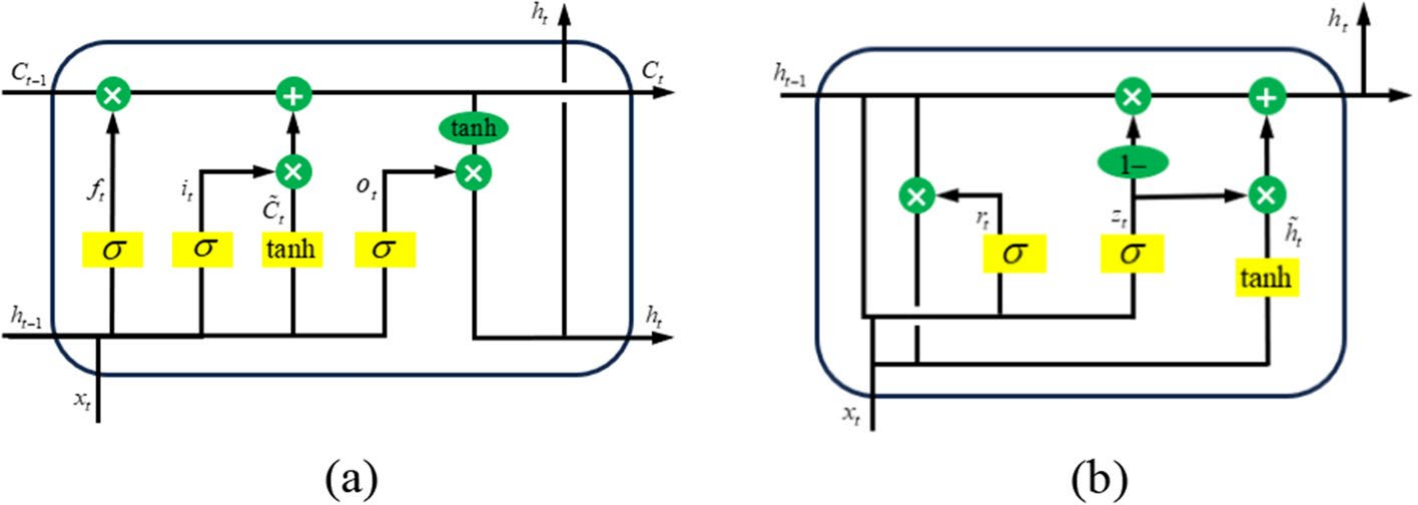
(**a**) Network structure diagram of LSTM in the first panel; (**b**) Network structure diagram of GRU in the second panel.

Gated Recurrent Unit is a kind of Recurrent Neural Network (RNN) and a variant of Long Short-Term Memory (LSTM). GRU can effectively deal with the problem of gradient disappearance and long term dependence. It simplifies the structure of LSTM, and controls the fusion degree of the current input with the previous hidden state and the forget-ting of the previous state by combining the input gate and the forget gate into an update gate. Compared with LSTM, GRU reduces one gating unit, so that fewer parameters are required for training, which can greatly improve the training efficiency and reduce the resources required for calculation^14^. Figure 1 shows the network structure of GRU and LSTM.

The $r_{t}$ and $z_{t}$ in Figure (b) represent reset gate and update gate, respectively. The reset gate controls the influence degree of the previous hidden state in the calculation of the candidate hidden state, and determines the contribution degree of the previous hidden state to the calculation of the candidate hidden state by adjusting the size of $r_{t}$. The update gate controls the update degree of the current input information to the hidden state. Through the adjustment of $z_{t}$ size, it decides how much information of the previous hidden state is retained and how much information of the current input is received. The reset gate and update gate are calculated as follows:

1 $$r_{t} = \sigma (W_{r} \cdot [h_{t - 1} ,x_{t} ])$$

2 $$z_{t} = \sigma (W_{z} \cdot [h_{t - 1} ,x_{t} ])$$

In the equation, $w_{r}$ and $w_{z}$ represent the weight of reset gate and update gate respectively, $h_{t - 1}$ represent the previous hidden state, $x_{t}$ represent the current input, the value range of $r_{t}$ and $z_{t}$ are both [0,1], and their size is determined by σ, which is the activation function sigmoid.

After the reset gate $r_{t}$ is obtained by Eq. (1), the hidden state $h_{t - 1}$ at the previous moment needs to be reset to obtain the candidate hidden state, which is calculated by the following formula:

3 $$\tilde{h}_{t} = \tanh (W_{c} \cdot [r_{t} * h_{t - 1} ,x_{t} ])$$

In the equation, $w_{c}$ is the weight of candidate hidden states.

After obtaining the update gate $z_{t}$ through Eq. (2), calculate the current hidden state $h_{t}$, and the formula is as follows:

4 $$h_{t} = (1 - z_{t} ) * h_{t - 1} + z_{t} * \tilde{h}_{t}$$

The first term in the equation represents the part that keeps the previous hidden state, and the second term represents the part that adopts the candidate hidden state.

### Deep Q-network 

#### Deep Q-network and double deep Q-network

DQN (Deep Q-Network) is a reinforcement learning algorithm based on deep learning, which is used to solve the Markov decision process of discrete action space. It uses neural networks to approximate the Q-value function, stabilizing the training process through techniques such as experience replay and target networks. In 2013, Minh et al.^15^ first proposed the Deep Q-network model, which is a convolutional neural network trained with a variant of Q-learning and successfully learns control policies directly from high-dimensional sensory inputs. In 2016. Silver et al.^16^ applied deep reinforcement learning algorithm to Go game and introduced Monte Carlo tree search method. Based on this algorithm, they wrote the program AlphaGo. In March of the same year, AlphaGo defeated Go world champion Lee Sedol 4–1, becoming the first artificial intelligence robot to directly defeat a human player.

In the original DQN algorithm, because the original algorithm uses the method of adopting the new strategy, the Q value is prone to be overestimated. Each time when the algorithm learns, it does not use the real action used in the next interaction, but the action with the maximum value considered by the current policy, resulting in the maximization bias, which makes the Q value of the estimated action too large.

In order to solve this problem, by decoupling action selection and value estimation, Hasselt et al.^17^ proposed the Double Deep Q-network (DDQN). Compared with DQN algorithm, DDQN algorithm changes the calculation method of the target value, which makes the training process more stable, can effectively solve the overestimation problem of the latter for the action value, and improve the convergence and performance of the algorithm.

DDQN constructs two action-value neural networks based on the experience replay mechanism and the skill of the target network. One is used to estimate the action and the other is used to estimate the value of the action. Instead of directly selecting all possible Q-values calculated by the target network by maximization, the DDQN algorithm first selects the action corresponding to the maximum Q-value by evaluating the network.

5 $$a = \mathop {\arg \max Q\left( {s_{t + 1} ,a;w_{e} } \right)}\limits_{a}$$

Then, the action a selected by the evaluation network is fed into the target network to calculate the target value. Therefore, it is only necessary to replace the method for computing the label value in the DQN algorithm (Eq. 6) by:

6 $$y_{t} = r_{t + 1} + \gamma Q(s_{t + 1} ,\mathop {\arg \max }\limits_{a} Q(s_{t + 1} ,a;w_{e} );w_{t} )$$

In the equation, y_t_ represents the target value, w_e_ represents the evaluation network parameters, and w_t_ represents the target network parameters.

#### Dueling deep Q-network and D3QN

Dueling DQN algorithm is an extension of traditional DQN, which includes a new neural Network structure, Duel Network^18^. The input of the competition network is the same as the input of DQN and DDQN algorithms, which is state information, but the output is different. Dueling DQN decomposes the estimation of the value function into state values and action advantages by introducing a baseline network and a dominance network. The state value represents the expected reward at a given state, while the action advantage represents how good each action is relative to the mean. With this decomposition, Dueling DQN can focus its attention on learning the state values instead of computing the values separately for each action. This makes the algorithm more efficient and better able to handle environments with large action Spaces. The evaluation network structure of Dueling DQN is as follows:

7 $$Q(s,a;w_{e} ) = V(s;w_{V} ) + A(s,a;w_{A} ) - mean_{a} A(s,a;w_{A} )$$

In the equation, V is the optimal state value function, A is the optimal advantage function, and the third term is the advantage function in the case of the average value of action a, which is generally 0.

In order to further reduce the overestimation of action values in the learning process, Wang et al. proposed an algorithm combining Double DQN and Dueling DQN algorithm, called D3QN^19^. The D3QN algorithm combines the idea of Double DQN and Dueling DQN algorithm to further improve the performance of the algorithm. The only difference between it and the Dueling DQN algorithm is the way in which the objective value is calculated. The calculation method of the target network is inspired by the idea of Double DQN, that is, the evaluation network is used to obtain the action corresponding to the optimal action value in the s_t+1_ state, and then the target network is used to calculate the action value of the action, so as to obtain the target value.

The traditional DQN and D3QN algorithms often use the hard update method to update the target network parameters. In Eq. (6), every interval C steps, the parameters of the evaluation network $w_{e}$ will be passed to the parameters of target network $w_{t}$. The larger the target network update interval C is, the more stable the algorithm will be. The slower the target network update frequency is, the slower the algorithm convergence speed will be. An appropriate target network update interval C can make the training of DQN algorithm both stable and fast. This update method of replacing the network parameters as a whole is called hard update, and it is often used in DQN and its later improved algorithms.

## SU-D3QN-G combinational model

### Model introduction and innovation

**Figure 2:**
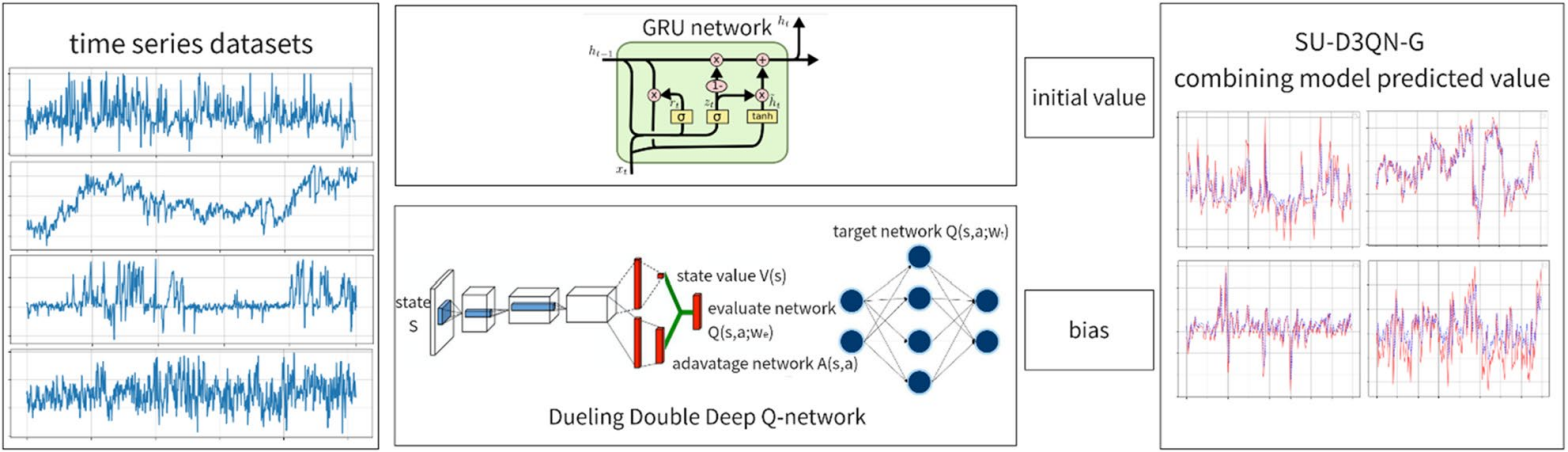
Algorithm structure.

The SU-D3QN-G model is composed of two algorithms, SU-D3QN and GRU. As a variant of RNN, GRU is easier to alleviate the vanishing gradient problem during training and can better capture long-term dependencies. Since GRU has a small number of parameters, it is less prone to overfitting on small datasets and can show stronger generalization ability compared to LSTM. As an upgrade of Double DQN and Dueling DQN, SU-D3QN has the advantages of both. The traditional DQN algorithm is easy to overestimate the Q value, which may lead to instability and performance degradation during training. Double DQN significantly alleviates the over-estimation problem by separating the selection and evaluation of Q-values. Dueling DQN introduces the concept of state value function and dominance value function. The state value function estimates the expected value of the state, while the dominance value function estimates the advantage of each action over other actions. This decomposition enables Dueling DQN to better understand the relationship between state values and action advantages, which helps to improve the stability and performance of the algorithm. The combination of the two can improve the learning speed of SU-D3QN and make the training process more stable. The algorithm structure diagram of this paper is shown in Fig. 2.

The D3QN algorithm used by previous scholars often uses a hard update method to update the target network and the evaluation network. For the time series prediction of leaf wire making links, this research uses SU-D3QN based on soft update strategy to update the parameters of the evaluation network. The target network of SU-D3QN is completely consistent with the model and parameters of the evaluation network. Every time the parameters of the evaluation network are updated, the parameters of the target network are also updated. Soft update smoothens the update process of the target network by mixing the parameters of a small part of the evaluation network with those of the target network at each update. This mixing ratio is controlled by the hyperparameter tau. The specific update formula is as follows:

8 $$w_{t} = tau*w_{e} + (1 - tau)*w_{t}$$

Soft updates can alleviate the instability problem in reinforcement learning algorithms. Although the learning speed may be relatively slow compared to hard updates, soft updates provide a smooth and gradual way of updating during the training process, which can increase the stability of the algorithm.

This research also introduces transfer learning. After max–min normalization of the data of the enterprise, this research uses the neural network trained in the previous stage to update the parameters of the next stage task. Since the source model has already been trained on the source task, it can save a lot of training time and computational resources.

**Figure 3:**
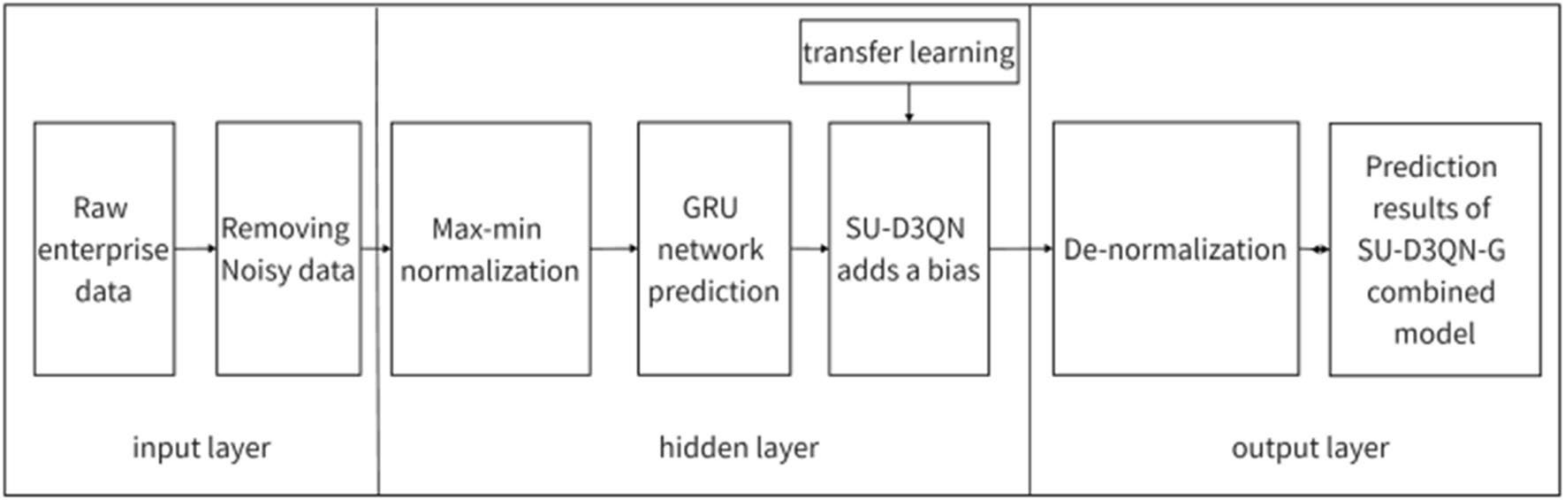
Algorithm flow chart.

Firstly, based on the raw data produced by the enterprise, after preprocessing, this research use GRU to predict it. Next, this research uses a reinforcement learning algorithm to learn and fit the bias at each time node based on the GRU model. The absolute error of SU-D3QN-G is further reduced. This bias is different for each time node and is learned by the algorithm. Through several training iterations, the average reward function curve of the SU-D3QN-G model converges on the training set. his research use the trained neural network model to check on the validation set and the test set. Figure 3 shows the algorithm flow of this paper.

### Algorithm design

The algorithm design mainly includes the state space, action space, reward function and the update method of the target network of reinforcement learning.

1. State space. The state space in this paper refers to the information observed before the true predicted value is derived. This research defines the state space as a set of length 7, which includes the predicted value of GRU at time t, the predicted value and the true value of GRU at time t-1, the predicted value and the true value of GRU at time t-2, and the predicted value and the true value of GRU at time t − 3.
2. Action space. The elements of the 7 dimensions of the state space are fed into the neural network, and the value of the bias of the different values at that time t is obtained. These biases, or corrections, make up the action space of the model. The agent selects the bias with the highest value, and on the basis of the predicted value of GRU at time t, the bias is added to obtain the predicted value of the SU-D3QN-G combined model.

The reward function. This research uses the mean absolute error index as the measurement index and choose the GRU algorithm as the benchmark. If the mean absolute error of the SU-D3QN-G model is lower than that of the original algorithm, a positive reward is obtained. The closer the combined model is to the true value, the larger is the absolute value of the reward. At the same time, this research multiplies a coefficient k to amplify the reward signal and reinforce the difference in payoff caused by different actions. The reward function is formulated as follows.

9 $$R = k\sum\limits_{i = 1} {\left( {AE_{i} - AE_{i}^{\prime } } \right)}$$

In the equation: k is the amplification factor; i is the length of the training set; AE is the absolute error of the original prediction sequence and AE' is the absolute error of SU-D3QN-G. This experiment sets the k value to 100 after preprocessing the data with max–min normalization. Table 1 shows the pseudo-code of the SU-D3QN algorithm.

**Table 1:** SU-D3QN algorithm pseudocode.

Algorithm Dueling Double Deep Q-network	
Input state s Output action a 1. Initialize the experience replay buffer D and the network parameter w 2. Set the current state s as the initial state 3. For each turn: Resets the current state s to the initial state 5. For each step in the round: Action a is selected using the epsilon-greedy strategy Perform action a and observe the next state s_t+1_ and reward r 8. Store the MDP(s, a, r, s_t+1_) into the experience replay buffer D 9. Randomly draw a batch of MDPS from D 10. For each MDP, compute the state value V and the dominance value A for each action 11. Calculate the action value of each action 12. Calculate the target action Q-value using the target network: 13. Compute the gradient descent based on the loss function: 14. Update the target network parameters w_e_ according to tau 15. Update state s to s_t+1_	

## Experiments, analysis and design of the system

### Experimental environment and data

#### Experimental environment 

The application algorithms in this paper are GRU and SU-D3QN, implemented based on Python3.9 language and Pytorch1.13 framework, configured with 12th Gen Intel(R) Core(TM) i9-12900H 2.50 GHz, RAM 16.0 GB. At the same time, LSTM and ARIMA were used for comparative experiments.

#### Experimental data preprocessing

**Figure 4:**
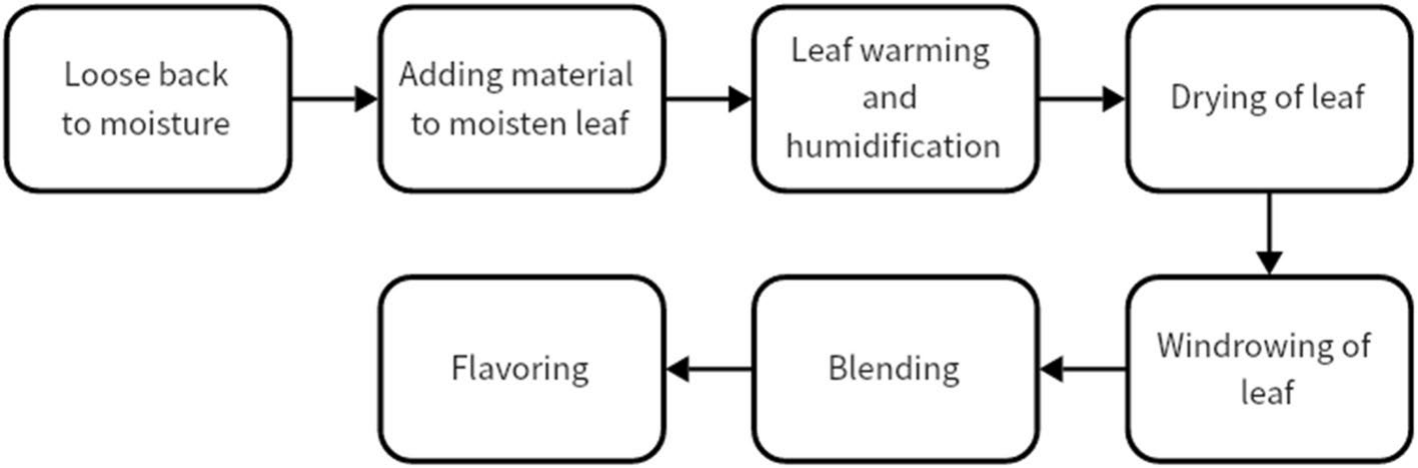
The silk production of multi-process.

In this experiment, the time series data of a silk production line of a company is used as the data set. The overall technological process of the production line includes: loose moisture recovery, adding material to moisten leaves, warming and humidifying leaves, drying silk, air selection of leaves, blending and flavoring. Among them, the four techno-logical processes from loose moisture recovery to silk drying improve the processing resistance of the leaf silk by adjusting the temperature and moisture content, so that the final product performs better in terms of taste and quality. Therefore, this experiment uses the time series data of four quality indicators: the outlet temperature of drying silk, the moisture of loose moisture return, the outlet temperature of feeding leaves, and the inlet water of leaf silk warming and humidification as data sets to verify the effectiveness of the proposed model in predicting the above indicators. Figure 4 illustrates the process of the multi-process production process of this enterprise.

**Figure 5:**
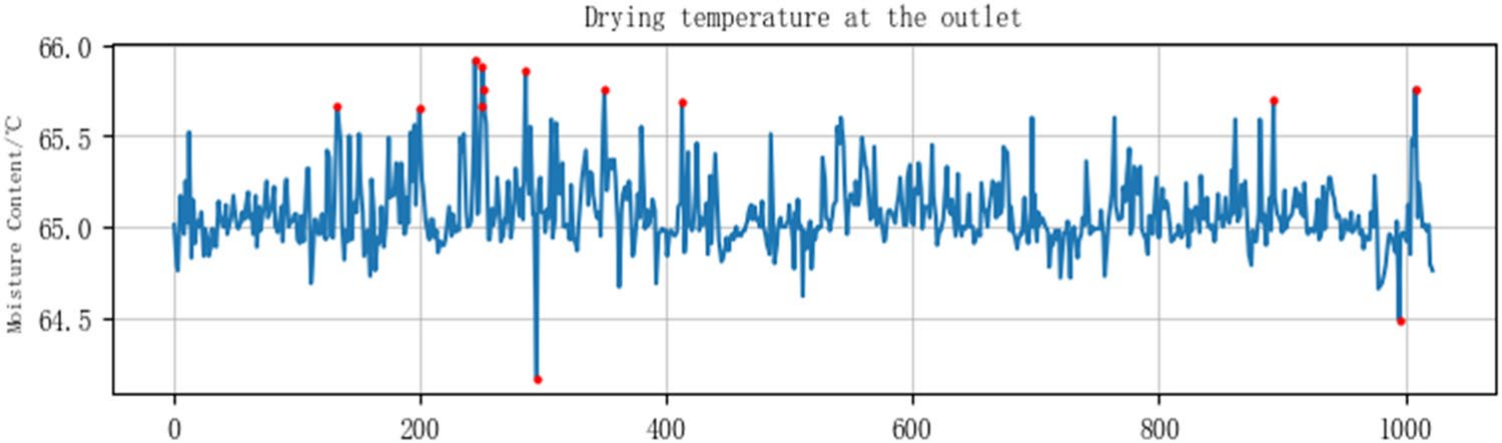
Anomalous data points.

**Figure 6:**
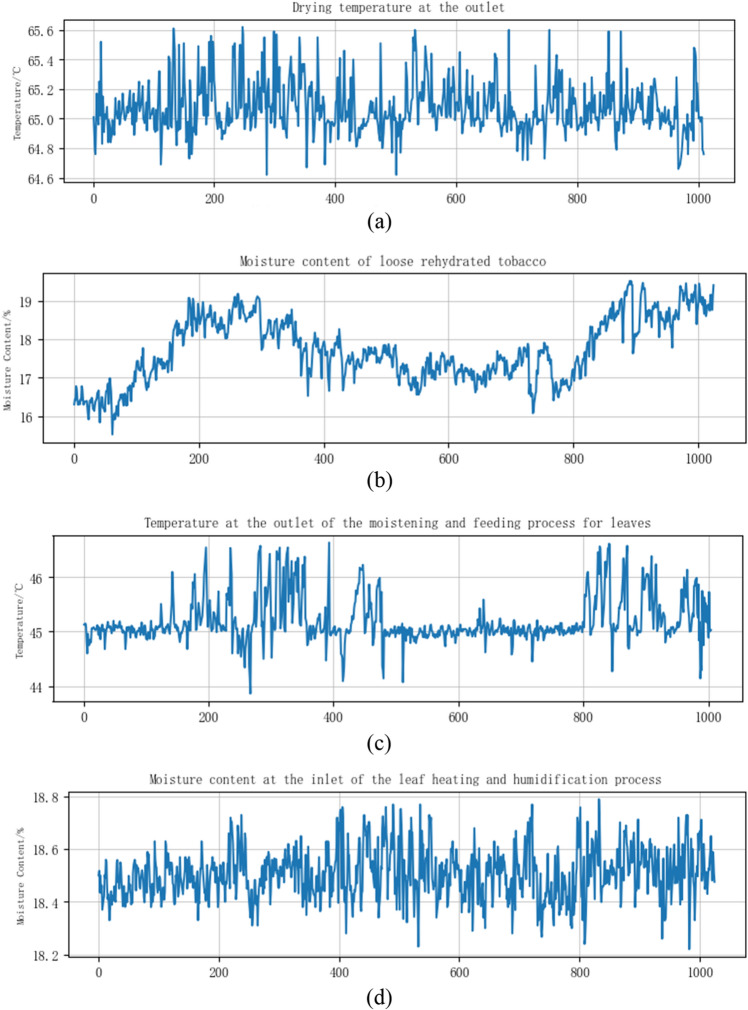
(**a**) Drying temperature at the outlet; (**b**) moisture content of loose rehydrated leaves; (**c**) temperature at the outlet of the moistening and feeding process for leaves; (**d**) moisture content at the inlet of the leaf heating and humidification process.

Due to the large differences in raw material composition between different production batches; In addition, during the opening and stopping operation of the production equipment, a large number of abnormal data will be generated, which leads to the characteristics of multi-noise data collected by the sensor. Firstly, the data were cleaned based on the production process specification. Then, criteria are used to further screen outliers to eliminate noise or abnormal interference in the data. Take the drying temperature at the outlet as an example, as shown in Fig. 5, the red data points in the figure are abnormal data, and Fig. 6 is the line chart of the processed data set.

In order to verify the effectiveness of the algorithm, 1000 samples were selected from each of the two experimental data sets and divided into training set, inspection set and test set according to the ratio of 6:2:2. At the same time, in order to avoid the dimensional differences between the data affecting the fitting effect of the model, the maximum and minimum normalization of the data was performed before the experiment, and the calculation method was as follows:

10 $$X_{scale} = \frac{{X - X_{\min } }}{{X_{\max } - X_{\min } }}$$

### Evaluation metrics and experimental parameters

#### Evaluation metrics

In order to scientifically and accurately evaluate the effectiveness of the algorithm, this experiment chooses to use MSE, RMSE, MAE and MAPE as evaluation indicators. These four indicators comprehensively consider multiple aspects of the time prediction model. MSE measures the squared average error and is insensitive to outliers. RMSE can measure the overall error size and is more sensitive to outliers. MAE measures the average error size and has little impact on outliers. MAPE, on the other hand, focuses on the relative error magnitude and reflects the ratio between the predicted error and the actual value. Through the comprehensive use of these indicators, this experiment can evaluate the performance of the time prediction model more comprehensively, and thus accurately evaluate the effectiveness of the algorithm. The calculation methods of the above four indicators are as follows: (11)—(14):

11 $$MSE = \frac{1}{n}\sum\nolimits_{i = 1}^{n} {\left( {y_{i} - y_{i}^{p} } \right)}^{2}$$

12 $$RMSE = \sqrt {\frac{1}{n}\sum\nolimits_{i = 1}^{n} {\left( {y_{i} - y_{i}^{p} } \right)}^{2} }$$

13 $$MAE = \frac{1}{n}\sum\nolimits_{i = 1}^{n} {\left| {y_{i} - y_{i}^{p} } \right|}$$

14 $$MAPE = \frac{100\% }{n}\sum\nolimits_{i = 1}^{n} {\left| {\frac{{y_{i} - y_{i}^{p} }}{{y_{i} }}} \right|}$$

In the equation, n is the number of samples, $y_{i}$ is the actual value of sample i, and $y_{i}^{p}$ is the predicted value of sample i.

#### Experimental parameters

In terms of recurrent neural network algorithm, in the prediction model of GRU algorithm, a GRU layer and a fully connected layer are constructed. In the GRU layer, the number of layers is set to 2, the hidden layer (that is, the number of neurons) is set to 256, the learning rate is 0.001, the number of features is set to 1, the sampling batch is set to 32, and Adam is selected as the optimizer. In order to transform the output of the GRU model to be consistent with the dimensions of the prediction task, a fully connected layer is used at the end to perform linear transformation and dimension transformation, and the number of layers is 1.

The hyperparameters of the GRU algorithm are set as Table 2.

**Table 2:** Hyperparameter Settings of GRU algorithm.

Hyperparameter names	Values
Timestep	1
Layers	2
Hidden size	256
Feature size	1
Learning rate	0.001
Batch size	32
Episodes	50

In terms of reinforcement learning algorithm, the evaluation network of SU-D3QN has a four-layer network structure. The first layer of the algorithm is the input layer with a total of seven nodes, corresponding to the set of state Spaces of length seven. The second and third layers of the algorithm are the hidden layers, which are both fully connected layers with 256 neurons and activated by the ReLU function. The fourth layer of the algorithm is the output layer, the output layer outputs the value evaluation of each action, and the output dimension will change with the change of the state space. Based on the value of each action, the agent chooses the action with the highest value to take the next action. This research uses the Adam optimizer for gradient descent.

The hyperparameters of the SU-D3QN algorithm are set as Table 3.

**Table 3:** Hyperparameter settings of SU-D3QN algorithm.

Hyperparameter names	Values
State dimension	7
Action dimension	Varies with the dataset
Fully connected layers dimension	256
α	0.001
γ	0.99
tau	0.005
ε-initial	0.1
ε-end	0.05
Decay rate	0.001
Batch max size	1,000,000
Batch size	256
Episode	200

This research uses the long short-term memory network algorithm in the recurrent neural network for comparative experiments. In the LSTM prediction model, two LSTM layers are constructed. In the first LSTM layer, the number of neurons is set to 256, and the parameter of return sequences is set to True. In the second LSTM layer, the number of neurons is also set to 256, and the parameter of return sequences is set to the default value of False. The time step of the LSTM layer is set to 1, the learning rate is 0.001, the sampling batch is set to 32, and Adam is selected as the optimizer. A Dropout layer is built after each LSTM layer, and the regularization ratio is set to 0.2. Finally, a fully connected layer with the number of layers 1 is used to perform the linear transformation and dimensional transformation.

The hyperparameters of the LSTM algorithm are set as Table 4.

**Table 4:** Hyperparameter settings of LSTM algorithm.

Hyperparameter names	Values
Timestep	1
Layers	2
Units	256
Activation function	tanh
Dropout rate	0.2
Learning rate	0.001
Batch size	32
Episodes	100
Timestep	1
Layers	2
Units	256
Activation function	tanh

In addition, the combined model is compared with the ARIMA algorithm. When making predictions with ARIMA, the data is first divided; Then, the auto_arima function in pmdarima library is used to automatically select the best ARIMA model parameters p, d and q. In addition, ARIMA model is fitted with training set. Finally, the test set is used to make predictions and the prediction error is calculated.

### Experiment and analysis

The experimental indicators of the data set of a company include: (1) Drying temperature at the outlet; (2) Moisture content of loose rehydrated leaves; (3) Temperature at the outlet of the moistening and feeding process for leaves; (4) Moisture content at the inlet of the leaf heating and humidification process.

**Figure 7:**
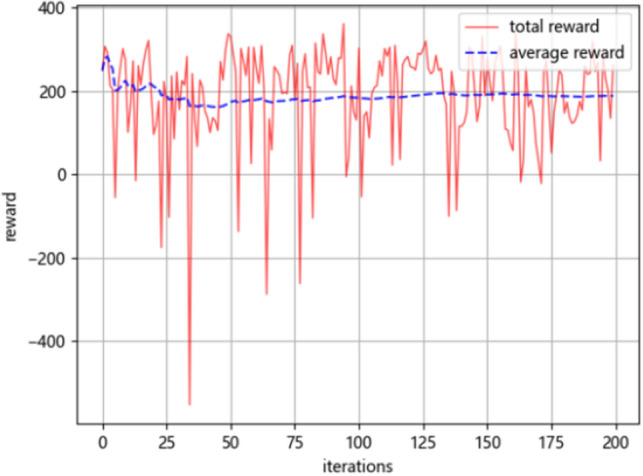
SU-D3QN-G model training reward curve.

Figure 7 shows the training curve of the SU-D3QN-G model on the training set of drying temperature at the outlet. The red solid line is the reinforcement learning total reward curve, and the blue dashed line is the average reward change curve. At the beginning of the iteration, the total reward curve fluctuates greatly because the initial value of the Ɛ-greedy strategy is large. As the number of iterations increases, the average reward curve gradually rises and converges after 150 iterations. Due to the difference of experimental data sets and the adjustment of model state space, the average reward value of different data sets may be different, but the average reward curve basically shows the change characteristics of first down, then up, and then tends to be stable. In this experiment, the number of training iterations for the four datasets is set to 200. After many experiments, it is found that the number of iterations of 200 times can better ensure the optimization of neural network parameters. Too few iterations make it difficult to learn features from the data, and too many iterations lead to overfitting on the training set, both of which can make the model perform worse on the test set.

**Figure 8:**
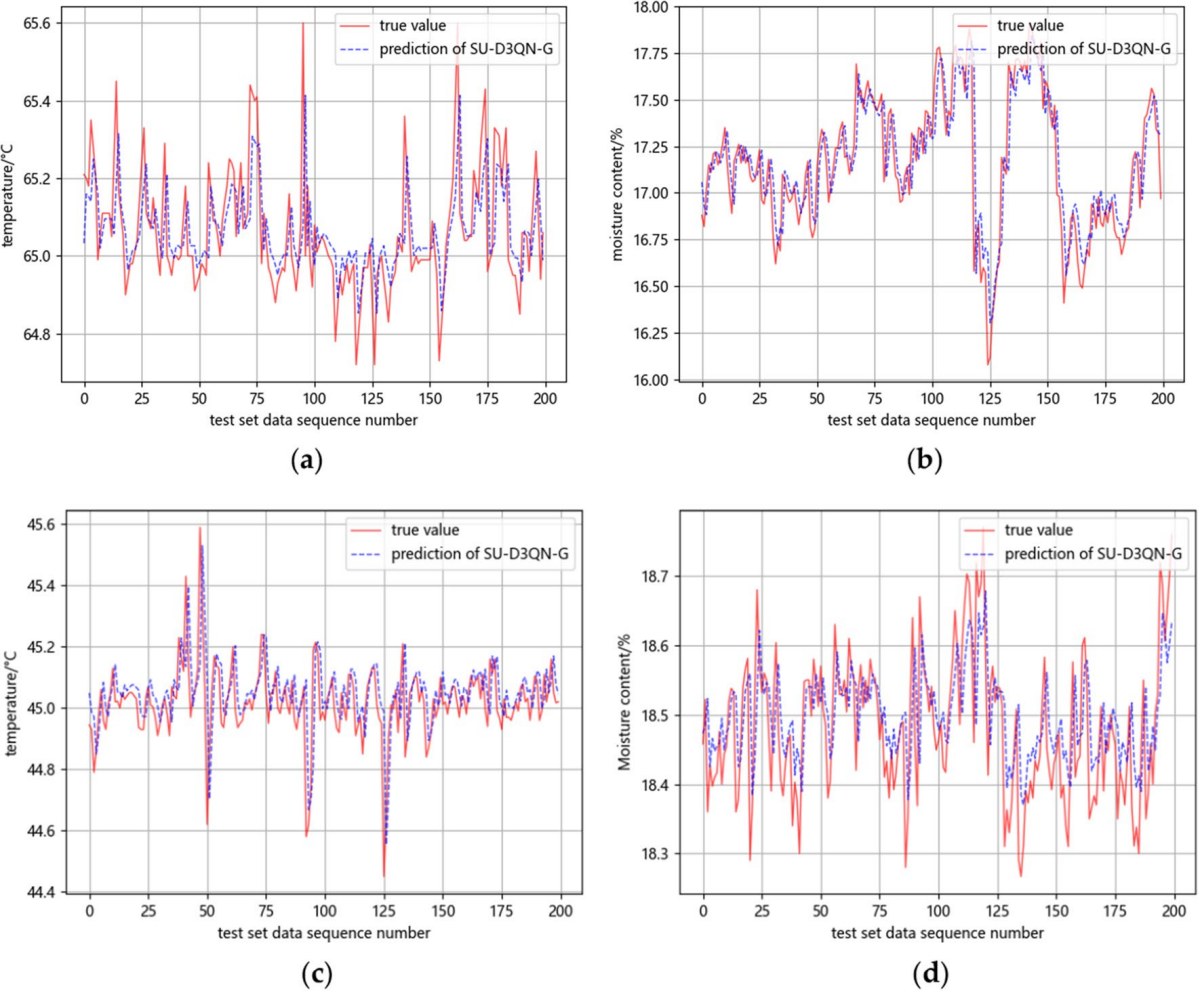
(**a**) Prediction of drying temperature at the outlet in the first panel; (**b**) prediction of moisture content of loose rehydrated leaves. (**c**) Prediction of temperature at the outlet of the moistening and feeding process for leaves; (**d**) prediction of moisture content at the inlet of the leaf heating and humidification process.

Figure 8 shows the predicted curve and the real value curve of the SU-D3QN-G combination model on the four data sets. According to the setup of the model, the nodes are predicted 200 times. From the figure, we can learn that the temperature data of the silk drying outlet fluctuates greatly, and the temperature data of the feeding leaves outlet fluctuates relatively little, so the combination model has a small improvement in the former, and a large improvement in the latter.

We summarized the prediction performance of the combined model, GRU, and LSTM. Table 5 shows the training set and test set performance of SU-D3QN-G, GRU and LSTM on the three data. Following the hyperparameter Settings in “Experimental parameters” section, we summarized the experimental results after training the combined model.

**Table 5:** Summary table of experimental results.

Data set	(1) Drying temperature at the outlet							
Test rating	SU-D3QN-G		GRU		LSTM		ARIMA	
	Training set	Test set	Training set	Test set	Training set	Test set	Training set	Test set
MSE	0.0221	**0.0140**	0.0220	0.0141	0.0222	0.0147	0.0214	0.0239
RMSE	0.1487	**0.1184**	0.1484	0.1189	0.1490	0.1214	0.1464	0.1547
MAE	0.1003	**0.0835**	0.1018	0.0852	0.1046	0.0906	0.1006	0.1251
MAPE	0.154%	**0.128%**	0.156%	0.131%	0.161%	0.139%	0.154%	0.192%

Data set	(2) Moisture content of loose rehydrated leaves							
Test rating	SU-D3QN-G		GRU		LSTM		ARIMA	
	Training set	Test set	Training set	Test set	Training set	Test set	Training set	Test set
MSE	0.0480	**0.0317**	0.0557	0.0360	0.0455	0.0434	0.0029	0.0429
RMSE	0.2190	**0.1781**	0.2360	0.1897	0.2132	0.2084	0.0543	0.2072
MAE	0.1661	**0.1217**	0.1856	0.1382	0.1574	0.1448	0.0414	0.1451
MAPE	0.945%	**0.711%**	1.050%	0.804%	0.897%	0.850%	0.835%	0.849%

Data set	(3) Temperature at the outlet of the moistening and feeding process for leaves							
Test rating	SU-D3QN-G		GRU		LSTM		ARIMA	
	Training set	Test set	Training set	Test set	Training set	Test set	Training set	Test set
MSE	0.0807	**0.0132**	0.0810	0.0163	0.0813	0.0157	0.0147	0.0146
RMSE	0.2841	**0.1148**	0.2846	0.1278	0.2852	0.1252	0.1212	0.1209
MAE	0.1682	**0.0758**	0.1729	0.0898	0.1738	0.0877	0.0812	0.0810
MAPE	0.370%	**0.168%**	0.381%	0.200%	0.383%	0.195%	0.382%	0.180%

Data set	(4) Moisture content at the inlet of the leaf heating and humidification process							
Test rating	SU-D3QN-G		GRU		LSTM		ARIMA	
	Training set	Test set	Training set	Test set	Training set	Test set	Training set	Test set
MSE	0.0049	**0.0064**	0.0057	0.0076	0.0055	0.0073	0.0012	0.0072
RMSE	0.0702	**0.0798**	0.0756	0.0873	0.0739	0.0854	0.0345	0.0846
MAE	0.0514	**0.0599**	0.0576	0.0673	0.0562	0.0662	0.0272	0.0655
MAPE	0.2774%	**0.324%**	0.3117%	0.365%	0.304%	0.359%	0.274%	0.354%

Bold indicates the performance of the SU-D3QN-G combination model on the test set.

Tables 6 and 7 show the analysis of the experimental results on the test set. In the second column of both tables, the combined model is not compared to itself, so the data in that column is null.

**Table 6:** Table of experimental results analysis in test set (absolute value and percent).

Data set	(1) Drying temperature at the outlet							(2) Moisture content of loose rehydrated leaves						
Reduction compared to the combined model	SU-D3QN-G	GRU		LSTM		ARIMA		SU-D3QN-G	GRU		LSTM		ARIMA	
MSE	–	0.0001	**0.846%**	0.0007	**5.132%**	0.0099	**70.714%**	–	0.0043	**13.451%**	0.0117	**36.920%**	0.0112	**35.331%**
RMSE	–	0.0005	**0.605%**	0.0030	**2.484%**	0.0363	**30.659%**	–	0.0116	**6.101%**	0.0303	**14.542%**	0.0291	**16.339%**
MAE	–	0.0017	**3.078%**	0.0071	**7.946%**	0.0416	**49.820%**	–	0.0165	**11.933%**	0.0231	**15.974%**	0.0234	**19.228%**
MAPE	–	0.003%	**3.098%**	0.011%	**7.987%**	0.0006	**50.000%**	–	0.093%	**11.559%**	0.139%	**16.395%**	0.0014	**19.409%**

Data set	(3) Temperature at the outlet of the moistening and feeding process for leaves							(4) Moisture content at the inlet of the leaf heating and humidification process						
Reduction compared to the combined model	SU-D3QN-G	GRU		LSTM		ARIMA		SU-D3QN-G	GRU		LSTM		ARIMA	
MSE	–	0.0032	**23.930%**	0.0025	**18.939%**	0.0014	**10.606%**	–	0.0013	**16.536%**	0.0009	**12.22%**	0.0008	**12.500%**
RMSE	–	0.0130	**10.118%**	0.0104	**8.304%**	0.0061	**5.314%**	–	0.0075	**8.642%**	0.0056	**6.54%**	0.0048	**6.015%**
MAE	–	0.0140	**15.678%**	0.0119	**13.630%**	0.0052	**6.860%**	–	0.0074	**10.953%**	0.0063	**9.57%**	0.0056	**9.349%**
MAPE	–	0.031%	**15.700%**	0.027%	**13.657%**	0.0001	**7.143%**	–	0.040%	**11.082%**	0.035%	**9.73%**	0.0003	**9.259%**

Bold indicates the boost of the combined model over the comparison algorithm.

**Table 7:** Table of experimental results analysis (reduction range of percentage).

Average reduction (percentage)	SU-D3QN-G	GRU	LSTM	ARIMA
MSE	–	0.846–23.930%	5.132–36.920%	10.606–70.714%
RMSE	–	0.605–10.118%	2.484–14.542%	5.314–30.659%
MAE	–	3.078–15.678%	7.946–15.974%	6.860–49.820%
MAPE	–	3.098–15.700%	7.987–16.395%	7.143–50.000%

Based on the best and worst performance of the combined model in Table 6 on the test set of each data set, we summarized to obtain the performance range of the combined model and filled in Table 7. The experimental results show that in the three groups of experiments, SU-D3QN-G combined time series prediction model comparing with GRU, LSTM and ARIMA, the MSE index is reduced by 0.846–23.930%, 5.132–36.920% and 10.606–70.714%, respectively. The RMSE index is reduced by 0.605–10.118%, 2.484–14.542% and 5.314–30.659%. The MAE index is reduced by 3.078–15.678%, 7.94–15.974% and 6.860–49.820%. The MAPE index is reduced by 3.098–15.700%, 7.98–16.395% and 7.143–50.000%.

In summary, we conclude that in the research of leaf production quality prediction, compared with the time series prediction method based on deep learning, the SU-D3QN-G combination model can further improve the accuracy and precision of prediction, provide more reliable information for enterprise production and operation, so as to make advance planning and decision-making.

### Design of the system

**Figure 9:**
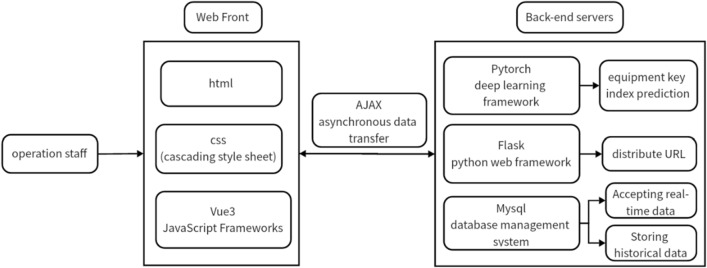
Design of real-time data analysis system based on time series database.

Based on the SU-D3QN-G combination model, a real-time data analysis system based on time series database is constructed for the prediction of various process indica-tors in leaf silk manufacturing. The design of the system is shown in Fig. 9:

Vue 3 is a popular open-source JavaScript framework for building user interfaces and single-page applications; AJAX is a technology for creating interactive Web applications; MySQL is a popular open-source relational database management system, which uses structured query language to manage and manipulate data. Flask is a lightweight Python Web framework for quickly building Web applications and apis. Vue3 is the front-end interactive software in this system. Based on the URL routing interface assigned by Flask, AJAX sends data to the backend through asynchronous transmission or returns it to the front end for page rendering after back-end calculation (Supplementary information).

**Figure 10:**
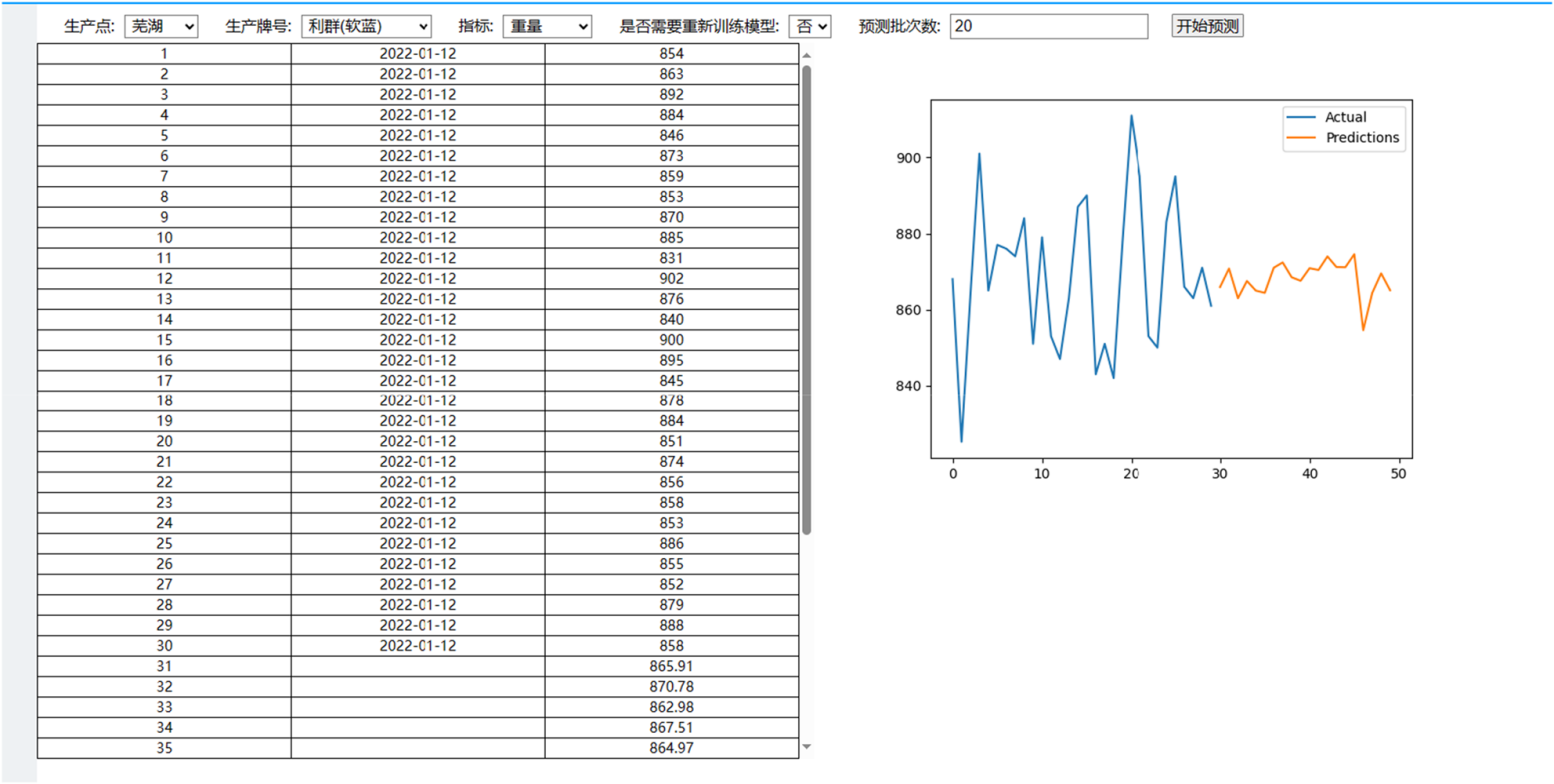
Application of combination model in information system of a company.

In the combined model, the training data of GRU and D3QN come from the database. Operators can select the relevant fields of the silk production link for retrieval to obtain the predicted values of the indicators for production decision-making, or call the latest data in the database to update the model through the “retrain” button of the interface. In general, the parameters of the combined forecasting model need to be updated once every six months to one year. In the practical application of the system, the SU-D3QN-G combined prediction model proposed in this paper reduces MSE, RMSE, MAE and MAPE by about 0.5–40% compared with the GRU prediction model used in the initial system. Figure 10 shows the prediction interface of a company's information system.

## Conclusions

In order to solve the problem of low prediction accuracy and poor stability of the current prediction model for the leaves wire making process indicators, this paper pro-poses a time series combination prediction model of SU-D3QN-G. From the test data of a company, the SU-D3QN-G combination model has a great improvement compared with the comparison algorithms in the four indicators of MSE, RMSE, MAE and MAPE, which verifies the effectiveness of the combination model in time series prediction. The experimental results show that the prediction model has less error in the process quality index prediction, and the prediction results are more accurate, which provides certain guiding significance for the process optimization and control decision-making of the silk production process. The main innovations and contributions of this paper are summarized as follows.

1. The SU-D3QN algorithm is combined with the time series prediction method GRU in the production process of leaf silk-making, and the key process parameters in leaf silk-making are predicted more accurately. This innovation breaks through the limitations of traditional prediction methods and improves the prediction accuracy and stability.
2. Introducing bias learning provides personalized adjustment for each time node, so that the model can better adapt to the complex changes and dynamic characteristics in silk production, and can learn data relationships that cannot be captured by a single time series prediction method.
3. The traditional SU-D3QN algorithm is improved. The evaluation network parameters of SU-D3QN are updated using soft update. This makes the performance of the model more stable during the training process and helps the convergence of the algorithm.
4. With the help of transfer learning, the neural network developed for the previous index is used for the training and prediction of the next index, which speeds up the learning speed of the agent and the convergence of the algorithm in reinforcement learning.

In the subsequent research, we will continue to incorporate the parameters that have a large impact on the quality indicators in the silk production process into the SU-D3QN-G model to achieve a more comprehensive and accurate prediction. On this basis, the real-time data analysis system based on time series database is improved to speed up the calling and training of the model. It is hoped that in the subsequent exploration, the control system can make more timely and accurate regulation decisions through the prediction results of the model, so as to improve the efficiency of the whole process and product quality.

## Data Availability

The data supporting the findings of this paper are available from the corresponding author upon reasonable request.
